# Autographa californica multiple nucleopolyhedrovirus *e18* is essential for the formation of normal intranuclear membrane microvesicles and intranuclear envelopment and nuclear egress of nucleocapsids

**DOI:** 10.1128/jvi.01338-25

**Published:** 2026-01-06

**Authors:** Lingqian Wang, Xiaowei Zhou, Xiyu Zhao, Xiaotao Zeng, Lu-Lin Li

**Affiliations:** 1 Hubei Key Laboratory of Genetic Regulation and Integrative Biology, College of Life Sciences, Central China Normal University 12446, Wuhan, China; Wageningen University & Research, Wageningen, Netherlands

**Keywords:** baculovirus, E18, intranuclear microvesicles, ODV envelopment, nuclear egress of the nucleocapsids, nuclear trafficking of envelope protein, low-complexity domain

## Abstract

Autographa californica multiple nucleopolyhedrovirus (AcMNPV) E18 (AC143, ODV-E18) is an envelope protein common to both occlusion-derived virions (ODVs) and budded virions (BVs). The *e18* gene has been demonstrated to be essential for generating infectious BVs. However, its functional role in virion morphogenesis remains unclear. In this study, we constructed an *e18* knockout virus and an *e18* repair virus to investigate the effects of *e18* deletion on virion morphogenesis. Our data indicated that *e18* is required for normal intranuclear microvesicle (IMV) formation and accumulation, for intranuclear envelopment and nuclear egress of nucleocapsids, as well as for embedding of ODVs into occlusion bodies (OBs) and BV production. Additionally, we created and characterized a series of recombinant viruses with truncated *e18* of varying lengths to identify domains involved in nuclear translocation and virion morphogenesis. We identified two low-complexity domains (LCDs) in E18, in addition to a known transmembrane domain (TM). The AA30-34 sequence within the TM was found to be essential, but not sufficient for nuclear translocation. However, an α-helix structure encompassing the TM domain proved adequate to mediate a fusion protein’s trafficking into the nucleus in the context of additional viral factors. Furthermore, we discovered that the TM was required for the accumulation of IMVs, while both the TM and LCD 1 were necessary for intranuclear envelopment, nuclear egress of nucleocapsids, and the embedding of ODVs into OBs; LCD 2 influenced the processing of IMVs and ODV formation. Both the TM and the two LCDs were essential for BV production.

**IMPORTANCE:**

The envelope protein E18 is a conserved component common to both ODV and BV virion types of baculoviruses, yet its functional role in virion morphogenesis remains unclear. This study investigated the *e18* gene of Autographa californica multiple nucleopolyhedrovirus, determining that it is essential for normal IMV formation and accumulation, intranuclear envelopment and nuclear egress of nucleocapsids, as well as for the embedding of ODVs into occlusion bodies and BV production. The functional roles of the single TM domain and two LCD domains within E18 during virion morphogenesis were identified. Furthermore, it was found that an α-helix structure encompassing the TM domain is sufficient to facilitate the trafficking of a fusion protein into the nucleus in the context of other viral factors, with AA30-34 being critical for the nuclear import of E18.

## INTRODUCTION

Baculoviruses are a group of viruses characterized by their large, single circular, double-stranded DNA genomes, which are packaged in enveloped, rod-shaped nucleocapsids. A striking feature of baculoviruses is the production of two structurally and functionally distinct virion phenotypes, namely, budded virus (BV) and occlusion-derived virus (ODV), during their infection cycle. Baculoviruses undergo DNA replication, transcription, and nucleocapsid assembly within the nucleus of infected cells. During morphogenesis, a portion of the nucleocapsids assembled in the nucleus penetrate the nuclear membrane, traverse the cytoplasm, and ultimately exit the cell by budding from the cytoplasmic membrane, forming BVs. These BVs derive their envelope from the modified plasma membrane. Once released from the infected cells, BVs spread the infection throughout the host insect’s body via hemolymph circulation. Meanwhile, the remaining nucleocapsids in the nucleus are enveloped by membranes derived from virus-induced intranuclear microvesicles (IMVs), positioned in a ring zone near the nuclear membrane, to form ODVs. These ODVs become embedded in large crystalline occlusion bodies (OBs). The release of ODV-containing OBs from infected insects contaminates food plants, thereby spreading the infection orally among host insect populations (1). The Autographa californica multiple nucleopolyhedrovirus (AcMNPV) belongs to the species *Alphabaculovirus aucalifornicae* in the genus *Alphabaculovirus* and is the most studied baculovirus.

BVs and ODVs, while having identical genomes and a shared nucleocapsid structure, show differences in their envelope’s origin and composition (2–5). Envelope proteins, key components of the viral envelope, perform numerous crucial functions in the virus infection and replication process. In AcMNPV, five proteins are specifically associated with the BV envelope, namely, GP64, GP37, AC75, AC93, and vUB1, whereas eleven proteins are unique to the ODV envelope, including PIF0-3, PIF6-9, ODV-E66, AC150, and GP41 (2, 6, 7). Each of these proteins plays distinct roles vital for their respective functions in the virus lifecycle. Ten proteins are common to both BV and ODV envelope, namely, ODV-E25, ODV-E18, BV/ODV-E26, AC23, AC76, AC78, AC92, AC103 (P48), PIF4, and PIF5 (2, 8–11). These proteins likely contribute to fundamental aspects of the virus’s life cycle. ODV-E25, ODV-E18, AC76, AC78, AC92, and P48 are known to be essential for generating infectious BVs (8–10, 12–15), while ODV-E25, AC76, and P48 are also necessary for ODV production (10, 12, 15).

The envelopment of ODV nucleocapsids is completed within the host cell’s nucleus; therefore, ODV envelope proteins must be transported into the nucleus from the cytoplasm, where they are synthesized during virus morphogenesis. A selection of ODV envelope proteins including ODV-E66, PIF1, PIF2, PIF3, PIF4, PIF8, Ac91, and Ac150 were discovered to contain an inner nuclear membrane-sorting motif (INM-SM). This motif consists of a hydrophobic domain of roughly 18 amino acids and a positively charged amino acid within 4–8 amino acids of the hydrophobic domain (16). ODV-E66 was the first INM-SM containing the ODV envelope protein to be studied; its N-terminal region was found to efficiently traffic fusion proteins to the nuclear envelope, IMVs, and the ODV envelope (17). The INM-SM sequence contained in this region was identified as being responsible for trafficking ODV-E66 from the endoplasmic reticulum (ER) to the inner nuclear membrane (18). Two viral proteins, FP25 and BV/ODV-E26, alongside a cellular protein, importing-α−16, were found to participate in this pathway (19, 20). Another subset of ODV envelope proteins, such as AC23, E18, AC76, AC78, PIF0, PIF5, PIF6, and PIF9, contain an atypical type of INM-SM. In this type, the TM can be located in the middle or near the C terminus of the protein, with associated positively charged amino acids located close to the N and/or C-terminal end of the transmembrane domain (21).

ODV-E18 was initially identified as an ODV envelope protein in AcMNPV and was subsequently found to be associated with the virus-induced intranuclear membrane (22). Given its presence in the BV envelope as well (4), it will be referred to as E18 in the remainder of this paper. *e18* is one of the thirty-eight “core genes” that possess homologs in all sequenced baculovirus genomes to date (13) and is among the most highly expressed genes in AcMNPV (23), suggesting it plays a crucial role in the virus life cycle. In AcMNPV, E18 is encoded by ORF 143, which is predicted to encode a peptide of 90 amino acids (22, 24). Notably, AcMNPV E18 features a hydrophobic TM in its central region (21). It has been reported that a recombinant bacmid of AcMNPV with *e18* knocked out fails to complete replication in transfected cells (13). However, the specific function of E18 in virus replication remains unclear. In this study, we conducted a functional analysis of AcMNPV E18. We created an *e18*-knockout AcMNPV recombinant bacmid and a set of recombinants with either a full-length or truncated *e18* translocated to the polyhedrin locus of the *e18*-knockout bacmid. We then investigated the subcellular distribution of both native and truncated E18, as well as the virus morphogenesis of these recombinants in transfected cells. Our experimental results indicate that E18 is essential for the production of ODVs and the nuclear egress of nucleocapsids. We discovered that the TM and two low-complexity domains (LCDs) identified in E18 play significant roles in the aggregation of IMVs and the envelopment of ODVs, respectively. Furthermore, we identified the sequence responsible for its nuclear translocation.

## RESULTS

### The deletion of *e18* resulted in an interruption in the reproduction of both BV and ODV

**Fig 1:**
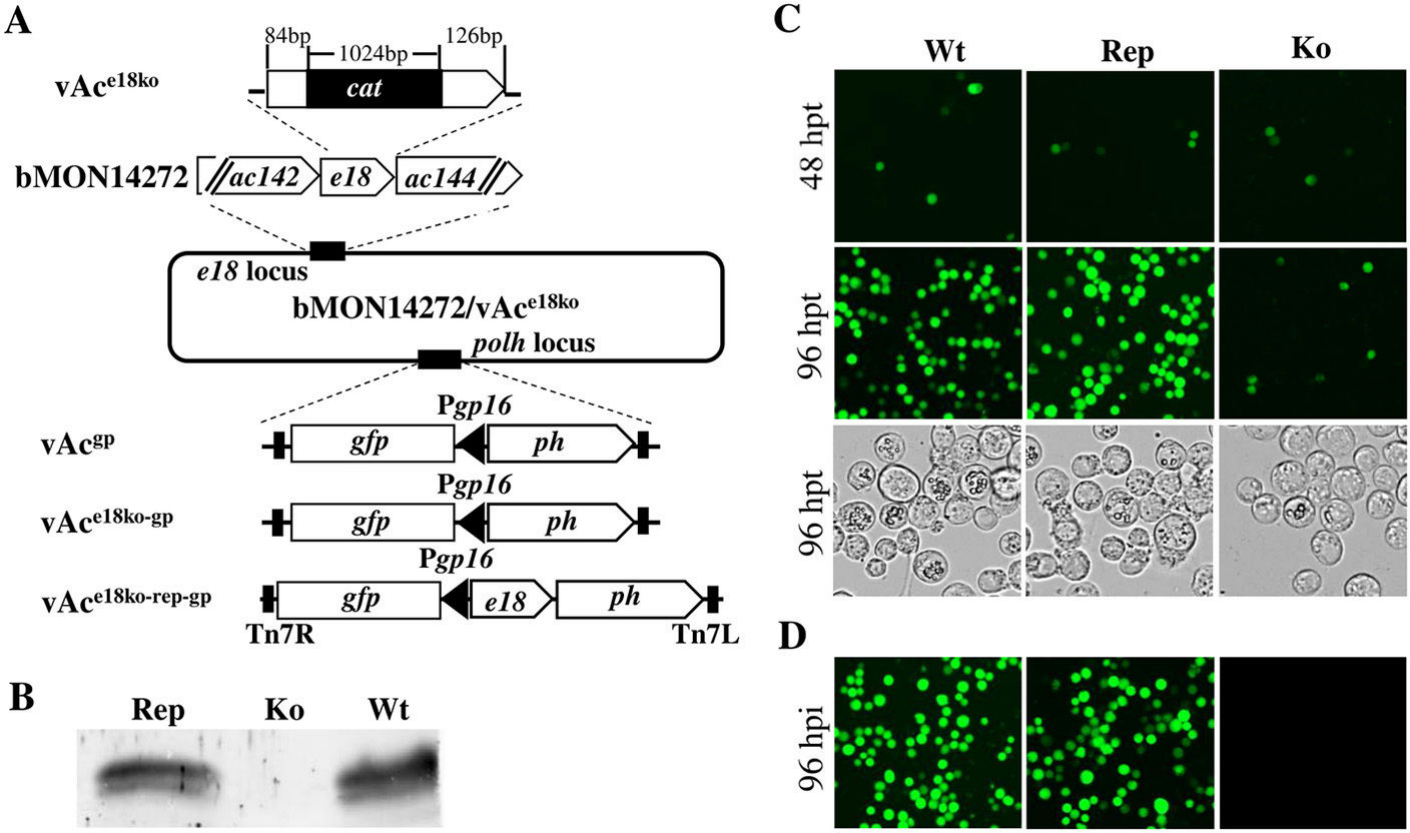
The construction and analysis of BV production of *e18* knockout, repair, and wild-type AcMNPV bacmids. (**A**) The maps illustrate the structures of the *e18* locus and the *polh* locus in the wild-type AcMNPV bacmid bMON14272, as well as in the recombinant bacmids. In the *e18* locus, a 60 nt sequence of the *e18* ORF was replaced with the *cat* gene to produce vAc^e18ko^. A copy of *egfp* under the control of the *gp16* promoter (Pgp16.gfp) and a copy of *polh* (*ph*) with its native promoter were inserted into the *polh* locus of bMON14272 or vAc^e18ko^ to generate vAc^gp^ and vAc^e18ko-gp^, respectively. A copy of *e18* with its native promoter and Pgp16.gfp and the *polh* was inserted into the *polh* locus of vAc*e18ko* to produce vAc^e18ko-rep-gp^. (**B**) Western blot analysis was conducted to detect the presence or absence of E18 in cell extracts transfected with vAc^e18ko-rep-gp^ (Rep), vAc^e18ko-gp^ (ko), or wild- type AcMNPV (Wt). (**C**) Fluorescence microscopy was performed on Sf9 cells transfected with vAc^gp^, vAc^e18ko-gp^, and vAc^e18ko-rep-gp^ at 48 hpt and 96 hpt. (**D**) Fluorescence microscopy was conducted on Sf9 cells inoculated with supernatants from transfections with vAc^gp^, vAc^e18ko-gp^, or vAc^e18ko-rep-gp^ at 96 hpt, and light microscopy was performed on the cells at 96 hpi.

The necessity of *e18* for virus replication has been previously established (13). To delve deeper into the role of *e18* in virus replication, we created an *e18* knockout AcMNPV bacmid mutant (vAc^e18ko-gp^), an *e18* knockout repaired bacmid (vAc^e18ko-rep-gp^), and a wild-type control bacmid (vAc^gp^), as detailed in the Materials and Methods section and illustrated in Fig. 1A and B. To gage the impact of the *e18* knockout on virus replication, the above-mentioned bacmids were individually transfected into Sf9 cells. Subsequently, the supernatants derived from these transfections were collected at 120 hours post-transfection (hpt) and used to infect fresh Sf9 cells. Fluorescence microscopy revealed some cells exhibiting fluorescence across all transfections at 48 hpt (Fig. 1C), suggesting successful bacmid introduction into the cells and viral gene expression. By 96 hpt, fluorescence and OBs were predominantly present in most cells infected with vAc^gp^ or vAc^e18ko-rep-gp^. In contrast, cells transfected with vAc^e18ko-gp^ exhibited fluorescence and OBs only in a limited fraction (Fig. 1C). Notably, dishes inoculated with the supernatant from the vAc^gp^ or vAc^e18ko-rep-gp^ transfections displayed nearly universal fluorescence and were filled with OBs at 96 hours post-infection (hpi). However, the dish inoculated with the supernatant from the vAc^e18ko-gp^ transfection showed neither fluorescence nor OBs (Fig. 1D), indicating an absence of infectious BV production in the transfected cells. These findings align with those of prior studies (13) and underscore that the knockout of *e18* halts BV production.

**Fig 2:**
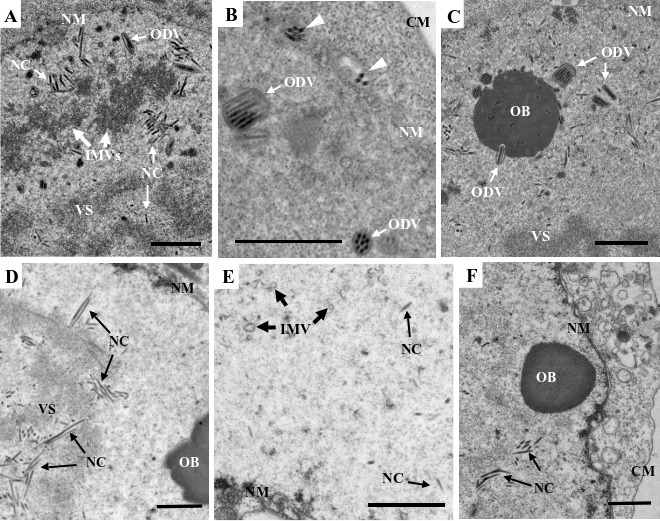
Transmission electron microscopy of Sf9 cells transfected with vAc^e18ko-rep-gp^ (**A–C**) or vAc^e18ko-gp^ (**D–F**) at 96 hpt. (**A**) Nucleocapsids within the VS, and a cluster of aggregated IMVs (indicated by white arrows) in the periphery region, where some nucleocapsids associate with the IMVs, and a few nucleocapsids are enveloped (ODV). (**B**) Two multiply enveloped ODV virions and one singly enveloped ODV virion are observed in the periphery region in the nucleus; and two membrane vesicles each containing multiple nucleocapsids (indicated by white triangles) are associated with the outer surface of the nuclear membrane. (**C**) An OB with some multiply or singly enveloped virions embedded or being embedded. (**D**) Nucleocapsids within the VS. (**E**) A few scattered IMVs (indicated by black thick line arrows). (**F**) An OB without a virion, in the periphery region of the nucleus. CM, cytoplasmic membrane; NM, nuclear membrane; NC, nucleocapsid; VS, virogenic stroma. The white and black fine line arrows indicate NCs or ODVs, respectively. The white and thick line arrows indicate IMVs. The scale bar equals 1 µm.

To assess the impact of the *e18* knockout on virus morphogenesis, thin sections of the cells transfected with vAc^e18ko-gp^ or vAc^e18ko-rep-gp^ were examined at 96 hpt using transmission electron microscopy. In both vAc^e18ko-gp-^ and vAc^e18ko-rep-gp^-transfected cells, virogenic stroma filled with rod-shaped nucleocapsids was evident in the nucleus (Fig. 2A and D). In vAc^e18ko-rep-gp^-transfected cells, numerous IMVs, nucleocapsids linked to these microvesicles, and enveloped nucleocapsids (or ODVs) were predominantly observed at the nuclear periphery (Fig. 2A and B). Additionally, nucleocapsids that had been released from the nuclear membrane (Fig. 2B) and OBs with enveloped nucleocapsids embedded within were identified (Fig. 2C). Conversely, in vAc^e18ko-gp^-transfected cells, only a few scattered small microvesicles were present (Fig. 2E), with no evidence of enveloped nucleocapsids or nucleocapsids outside the nucleus. Moreover, the OBs observed in these cells did not have any associated nucleocapsids (Fig. 2F). These findings suggest that the deletion of *e18* led to a decrease in IMV formation, thereby inhibiting the accumulation of these microvesicles and the envelopment of nucleocapsids. This deletion also hindered the egress of nucleocapsids from the nucleus and formation of ODVs and their incorporation into OBs.

### Subcellular localization of E18 in infected cells

**Fig 3:**
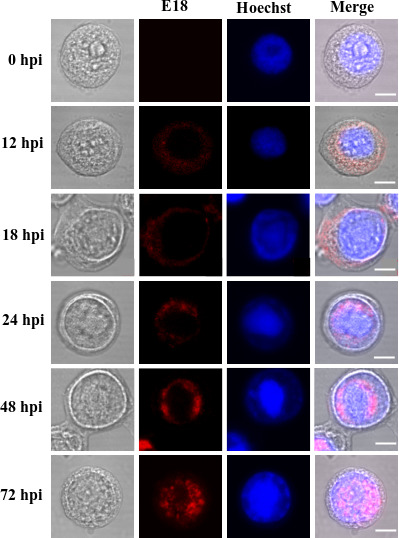
Subcellular localization of E18 in Sf9 cells infected with AcMNPV. The collection of Sf9 cells, infected with AcMNPV, was determined at specific intervals, namely, 0, 12, 18, 24, 48, and 72 hpi. The presence of E18 was identified through the use of E18-specific polyclonal antibodies. Subsequently, these were blotted with rhodamine-conjugated goat anti-rabbit IgG to label E18 (represented in red). Nuclear staining was achieved using Hoechst 33258 (represented in blue). The final observation of the cells was performed using confocal microscopy. The scale bar equals 5 µm. The scale bars in some of the IF images are replaced for better depiction and clarity.

It was demonstrated that E18 is associated with the envelopes of both BV and ODV, necessitating its transport into the nucleus from the cytoplasm where it is synthesized. To observe the dynamic distribution of E18 within infected cells, the subcellular localization of E18 in infected Sf9 cells at various time points post-infection was assessed using immunofluorescence microscopy. As depicted in Fig. 3, E18, labeled by rhodamine and producing red fluorescence, was initially observed in the cytoplasm at 12 hpi. By 18 hpi, a subtle red fluorescence was detectable in the nucleus, although predominantly still present in the cytoplasm. At 24 hpi, the red fluorescence was observed primarily in the nucleus, forming a peripheral ring zone. By 48 hpi, this ring zone extended toward the center of the nucleus. At 72 hpi, the red fluorescence-labeled E18 had permeated throughout the nucleus, with minimal presence in the cytoplasm. The results showed that E18 was highly expressed in the late stage of infection and transferred from the cytoplasm to the nucleus between 18 and 24 hpi. These correspond to the temporal expression and immunoelectron microscopic results previously reported (22).

### Preliminary mapping of E18 segments associated with nuclear translocation

**Fig 4:**
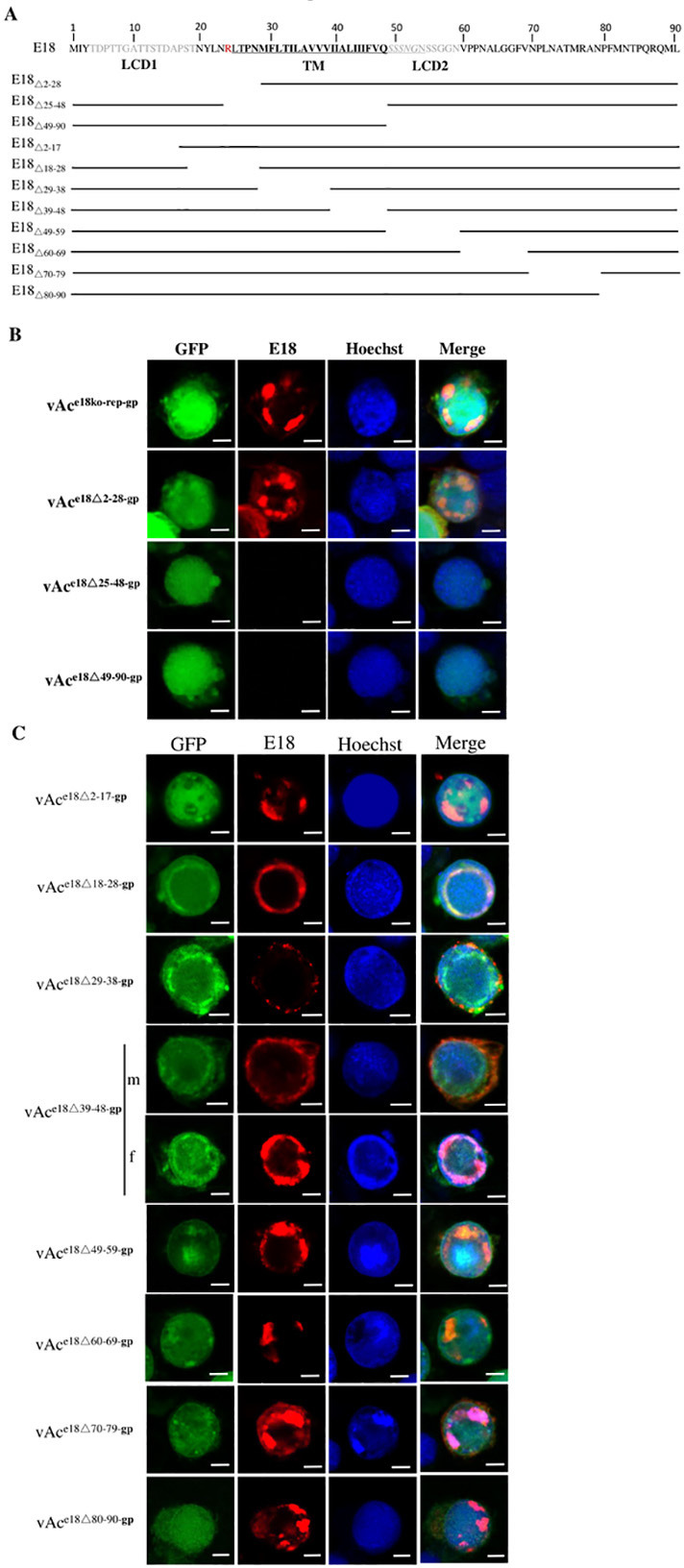
Preliminary mapping of E18 segments associated with nuclear translocation using immunofluorescence microscopy. (**A**) The entire amino acid sequence of AcMNPV E18 and a diagram of its truncations. The predicted α-helix structure sequence is underlined; the TM domain sequence is highlighted in bold, and LCD 1 and LCD 2 are in gray. E18_Δ2-28_, E18_Δ25-48_, E18_Δ49-90_, E18_Δ2-17_, E18_Δ18-28_, E18_Δ29-38_, E18_Δ39-48_, E18_Δ49-59_, E18_Δ60-69_, E18_Δ70-79_, and E18_80-90_ represent truncated E18 peptides designed for expression by recombinant bacmids vAc^e18Δ2-28-gp^, vAc^e18Δ25-48-gp^, vAc^e18Δ49-90-gp^, vAc^e18Δ2-17-gp^, vAc^e18Δ18-28-gp^, vAc^e18Δ29-38-gp^, vAc^e18Δ39-48-gp^, vAc^e18Δ49-59-gp^, vAc^e18Δ60-69-gp^, vAc^e18Δ70-79-gp^, and vAc^e18Δ80-90-gp,^ respectively. Construction of these recombinant bacmids is detailed in the Materials and Methods section. (**B**) Intracellular localization of E18 and the truncated E18 peptides in Sf9 cells transfected by vAc^e18ko-rep-gp^, vAc^e18Δ2-28-gp^, vAc^e18Δ25-48-gp^, and vAc^e18Δ49-90-gp^. (**C**) Intracellular localization of E18 and the truncated E18 peptides in Sf9 cells transfected by vAc^e18Δ2-17-gp^, vAc^e18Δ18-28-gp^, vAc^e18Δ29-38-gp^, vAc^e18Δ39-48-gp^, vAc^e18Δ49-59-gp^, vAc^e18Δ60-69-gp^, vAc^e18Δ70-79-gp^, and vAc^e18Δ80-90-gp^. Cells were sampled at 72 hpt. They were fixed and stained with E18-specific polyclonal antibodies and rhodamine-conjugated goat anti-rabbit IgG to label the truncated E18 peptides (red). The nuclei were stained with Hoechst 33258 (blue). Sixty cells showing red fluorescence were counted. The assays for each mutant were repeated at least twice. The photos shown in each row represent the subcellular distribution of the E18 or truncated E18 peptides in over 80% of the cells transfected with the individual bacmids indicated at the left side of each row, except those in (**C**) rows 4 & 5, where m and f indicate the phenotypes observed in 60% and 40% of the cells transfected with vAc^e18Δ39-48-gp^, respectively. The scale bar equals 5 µm. The scale bars in some of the IF images are replaced for better depiction and clarity.

A bioinformatic analysis on E18, conducted using SMART (https://smart.embl.de), TMHMM 2.0, and MemBrain (http://www.csbio.sjtu.edu.cn/bioinf/MemBrain/) software programs, predicted a putative TM comprising AA25-47/27-48, and two LCDs, one at the N-terminal (AA4-19) and the other immediately downstream of the TM (AA49-59) (Fig. 4A). In addition, AA27-54 is predicted to form an a-helix by using AlphaFold 2. No discernible nuclear localization signal (NLS) sequence was identified by using NLS-Mapper (https://nls-mapper.iab.keio.ac.jp/cgi-bin/NLS_Mapper_form.cgi).

To delineate the E18 sequence necessary for nuclear translocation, a series of recombinant AcMNPV bacmids featuring truncated *e18* were generated. Initially, three distinct recombinant bacmids vAc^e18Δ2-28-gp^, vAc^e18Δ25-48-gp^, and vAc^e18Δ49-90-gp^ were constructed and designed to express truncated E18 variants, namely, E18_Δ2-28_, E18_Δ25-48_, and E18_Δ49-90_, respectively (Fig. 4A). Figure 4B presents immunofluorescence microscopy images of Sf cells transfected with the individual recombinant bacmids or vAc^e18ko-rep-gp^ as a control, at 72 hpt. The cells transfected with vAc^e18ko-rep-gp^ or vAc^e18Δ2-28-gp^ displayed rhodamine-labeled E18 (row 1) or E18_Δ2-28_ (row 2), in the nucleus, respectively, whereas the cells transfected with vAc^e18Δ25-48-gp^ (row 3) or vAc^e18Δ49-90-gp^ (row 4) exhibited no fluorescence. These results indicate that AA2-28 is dispensable for nuclear translocation, while E18_Δ25-48_ and E18_Δ49-90_ were not detected at all.

Subsequently, eight additional recombinant bacmids—vAc^e18Δ2-17-gp^, vAc^e18Δ18-28-gp^, vAc^e18Δ29-38-gp^, vAc^e18Δ39-48-gp^, vAc^e18Δ49-59-gp^, vAc^e18Δ60-69-gp^, vAc^e18Δ70-79-gp^, and vAc^e18Δ80-90-gp^—were devised and analyzed, with the codons 2–17, 18–28, 29–38, 39–48, 49–59, 60–69, 70–79, and 80–90 of *e18* truncated, respectively (Fig. 4A). As shown in Fig. 4C, in the cultures transfected with the individual bacmids vAc^e18Δ2-17-gp^, vAc^e18Δ18-28-gp^, vAc^e18Δ60-69-gp^, vAc^e18Δ70-79-gp^, and vAc^e18Δ80-90-gp^, the truncated E18 variants E18_Δ2-17,_ E18_Δ18-28,_ E18_Δ60-69,_ E18_Δ70-79,_ and E18,_Δ80-90,_ which were labeled by rhodamine, were located in the nuclei of 95% of the transfected cells at 72 hpt (rows 1, 2, 7, 8, and 9). In all the cells transfected with vAc^e18Δ29-38-gp^, E18_Δ29-38_ was observed in the cytoplasm until 72 hpt (Fig. 4C row 3). In the cultures transfected with vAc^e18Δ39-48-gp^, or vAc^e18Δ49-59-gp^, E18_Δ39-48_ and E18_Δ49-59_ were present in the nuclei of 40% and 80% of the transfected cells, respectively (Fig. 4C rows 4 and 5, row 6). These results suggest that the AA29-48 region of E18 harbors the necessary sequence for nuclear translocation.

### The AA30-34 of E18 is essential for nuclear transport

**Fig 5:**
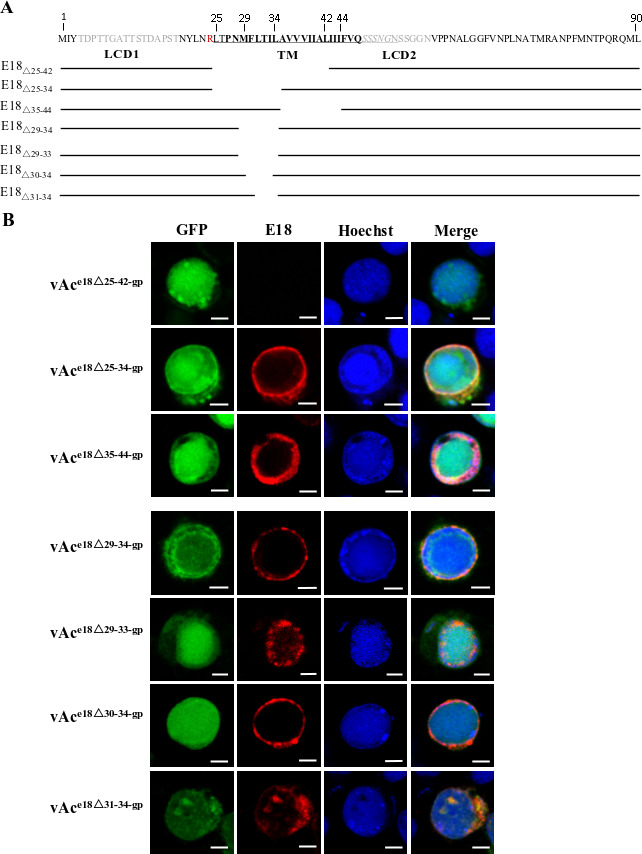
Identification of the E18 sequence crucial for nuclear translocation by immunofluorescence microscopy. (**A**) Schematic representation of AcMNPV E18 truncations. E18_Δ25-42_, E18_Δ25-34_, E18_Δ35-44_, E18_Δ29-34_, E18_Δ29-33_, E18,_Δ30-34,_ and E18_Δ31-34_ represent truncated E18 peptides designed for expression by recombinant bacmids vAc^e18Δ25-42-gp^, vAc^e18Δ25-34-gp^, vAc^e18Δ35-44-gp^, vAc^e18Δ29-34-gp^, vAc^e18Δ29-33-gp^, vAc^e18Δ30-34-gp^, and vAc^e18Δ31-34-gp^, respectively. Details on the construction of these recombinant bacmids can be found in the Materials and Methods section. (**B**) Intracellular localization of truncated E18 peptides in Sf9 cells transfected with the individual bacmids. Cells were sampled at 72 hpt. They were fixed and stained with E18-specific polyclonal antibodies and rhodamine-conjugated goat anti-rabbit IgG to label the truncated E18 peptides (red). The nuclei were stained with Hoechst 33258 (blue). Sixty cells showing red fluorescence were counted. The assays for each mutant were repeated at least twice. The photos shown in each row represent the subcellular distribution of the truncated E18 peptides in over 80% of the cells transfected with the individual bacmids indicated at the left side of each row. The scale bar equals 5 µm.

To validate the initial findings from immunofluorescence microscopy regarding the nuclear translocation sequence (NTS) and to precisely delineate the minimal peptide residues crucial for E18 nuclear transport, further truncations of E18 were created (Fig. 5A). Initially, three constructs were evaluated. As shown in Fig. 5B, truncated E18 was observed in the cytoplasm of all the cells transfected with vAc^e18Δ25-34-gp^ (row 2), in the nuclei of 95% of the cells transfected with vAc^e18Δ35-44-gp^ (row 3), but not in cells transfected with vAc^e18Δ25-42-gp^ (row 1). Subsequent evaluations of four additional constructs (vAc^e18Δ29-34-gp^, vAc^e18Δ29-33-gp^, vAc,^e18Δ30-34-gp,^ and vAc^e18Δ31-34-gp^) revealed that E18_Δ29-34_ and E18_Δ30-34_ were solely present in the cytoplasm of the cells transfected with vAc^e18Δ29-34-gp^ or vAc^e18Δ30-34-gp^ (rows 4 and 5). Conversely, E18_Δ29-33_ and E18_Δ31-34_ were identified in the nucleui of 80% and 95% of the cells transfected with vAc^e18Δ29-33-gp^ and vAc^e18Δ31-34-gp^, respectively (rows 6 and 7). Thus, the essential sequence for the nuclear transport of E18 was pinpointed to F_30_LTIL_34_ (Fig. 5A).

### The AA27-54 of E18 effectively mediates the trafficking of a fusion protein into the nucleus in the presence of additional viral factors

**Fig 6:**
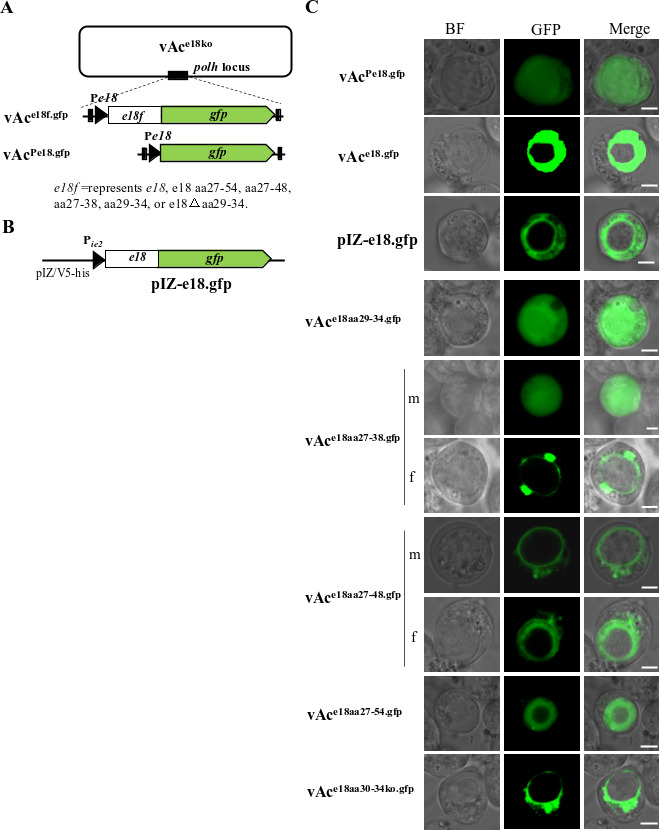
Identification of the E18 sequence sufficient to mediate a fusion protein trafficking into the nucleus by fluorescence microscopy. (**A**) Depicts the structures of the recombinant bacmids expressing E18 fragment-EGFP fusion proteins. A copy of *e18.gfp*, *e18aa27-54.gfp, e18aa27-48.gfp, e18aa27-38.gfp, e18aa29-34.gfp,* e18Δaa30-34.*egfp, or egfp* fusion gene sequence under control of an *e18* promoter was inserted into the *polh* locus of vAc^e18ko^, making vAc^e18.gfp^, vAc^e18aa27-54.gfp^, vAc^e18aa27-48.gfp^, vAc^e18aa27-38.gfp^, vAc^e18aa29-34.gfp^, vAc^e18Δaa30-34.gfp^, and vAc^Pe18.gfp^, respectively. (**B**) A copy of the *e18.gfp* fusion gene coding sequence was inserted downstream of the ie2 promoter of plasmid pIZ/V5-His, generating a transient expression plasmid pIZ-e18.gfp. (**C**) Intracellular localization of E18-EGFP, E18 fragment-EGFP fusion proteins, and EGFP in Sf9 cells transfected with the individual bacmids above. Cells were examined at 72 hpt. Sixty cells showing green fluorescence were counted. The assays for each mutant were repeated at least twice. The photos shown in each row represent the subcellular distribution of the E18- or truncated E18-EGFP fusion proteins, and EGFP in over 80% of the cells transfected with the individual bacmids indicated at the left side of each row, except those in (**C**) rows 4 and 5 and 6 and 7, where m and f indicate the phenotypes observed in 70% and 30% of the cells transfected with vAc^e18aa27-38gfp^ or in 60% and 40% of the cells transfected with vAc^e18aa27-48gfp^, respectively. The scale bar equals 5 µm.

To ascertain whether the peptide FLTIL functions as an NTS and to delineate the minimal sequence required for nuclear translocation, we constructed a series of *e18*-knockout bacmids: vAc^e18aa29-34.gfp^, vAc^e18aa27-38.gfp^, vAc^e18aa27-48.gfp^, vAc^e18aa27-54.gfp^, vAc^e18Δaa30-34.gfp^, vAc^e18.gfp^, and vAc^Pe18.gfp^. These vectors express fusion peptides E18_aa29-34_.GFP, E18_aa27-38_.GFP, E18_aa27-48_.GFP, E18_aa27-54_.GFP, E18Δaa30-34.GFP, E18.GFP, and GFP, respectively, all under the control of the *e18* promoter (Fig. 6A). Notably, previous studies have indicated that E18 cannot autonomously enter the nucleus (25). To validate this observation, we also constructed a plasmid expressing the E18-GFP fusion protein (Fig. 6B). Figure 6C shows the distribution of these fusion peptides and GFP in the cells transfected by the individual bacmids, at 72 hpt. The GFP expressed by vAc^Pe18.gfp^ was observed both in the cytoplasm and the nucleus in all the transfected cells (row 1). However, the fusion peptide E18.GFP in 97% of the cells transfected with vAc^e18.gfp^ was found in the nuclei (row 2), while the E18.GFP in all the cells transfected with the transient expression plasmid pIZ-e18.gfp remained exclusively in the cytoplasm (row 3). These findings suggest that the GFP can freely traverse the nuclear pore, while the E18.GFP requires additional viral factors for nuclear transport. In the transfection conducted with vAc^e18aa29-34.gfp^, the phenotype of the E18_aa29-34_.GFP mirrored that of the GFP within cells containing vAc^Pe18.gfp^ (row 4). This similarity suggests that the peptide FLTIL does not function as an NTS. In contrast, other bacmid constructs yielded different results in their respective cultures. Specifically, the E18_aa27-38_.GFP was observed to spread throughout the entire cell in most (70%) cells (row 5) or around the periphery of the nuclear membrane in a few (30%) cells (row 6) in the culture with vAc^e18aa27-38.gfp^. For the E18_aa27-48_.GFP, it was present around the outside of the nuclear membrane in most (60%) cells (row 7) and occupied the ring zone of the nuclei in few cells (row 8), as seen in the culture with vAc^e18aa27-48.gfp^. Surprisingly, E18_aa27-54_.GFP was located in the ring zone of the nuclei in 97% of the cells transfected by vAc^e18aa27-54.gfp^ (row 9), implying that AA27-54 has the potential to mediate efficient nuclear transposition. Finally, in the cells transfected by vAc^e18Δaa29-34.gfp^, the E18Δaa29-34.GFP remained exclusively in the cytoplasm (row 10), indicating that AA29-34 is also essential for nuclear import of the E18-EGFP fusion protein.

### The TM domain of E18 is essential for the accumulation of IMVs, as well as for the nuclear envelopment and the nuclear egress of nucleocapsids

**Fig 7:**
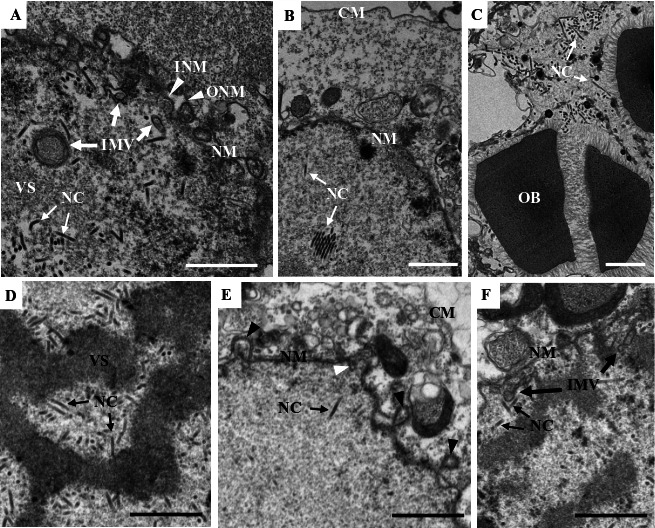
Transmission electron microscopy images of Sf9 cells transfected with vAc^e18Δ29-38-gp^ (**A–C**) or vAc^e18Δ39-48-gp^ (**D–F**) at 96 hpt. (**A**) Nucleocapsids within the VS, a few IMVs near the nuclear membrane, and some membrane microvesicles residing between the outer and inner nuclear membrane (indicated by white triangles). (**B**) A segment of a cell with a nucleocapsid near the nucleus, with no nucleocapsids appearing outside of or budding through the nuclear membrane. (**C**) A cracked OB without virions. (**D**) A part of VS with numerous nucleocapsids strapped. (**E**) A segment of a cell with some nucleocapsids in the periphery region in the nucleus, several microvesicles budding into the cytoplasm from the nuclear membrane (indicated by black triangles), and a microvesicle budding into the nucleoplasm (indicated by a white triangle). (**F**) Several IMVs near the nuclear membrane (indicated by black thick line arrows). CM, cytoplasmic membrane; NM, nuclear membrane; NC, nucleocapsid; VS, virogenic stroma; ONM, outer nuclear membrane; INM, inner nuclear membrane. The white and black fine line arrows indicate NCs. Scale bar = 1 µm.

To examine the effects of various truncations of E18 on virus morphogenesis, thin sections of the cells transfected with individual bacmids containing truncated *e18* were prepared at 96 hpt and examined by transmission electron microscopy. In the cells transfected with vAc^e18Δ29-38-gp^ or vAc^e18Δ39-48-gp^, which contain *e18* mutants with TM coding sequence truncated, virogenic stroma and nucleocapsids were present in the nucleus (Fig. 7A and D). Figure 7A is a section of the nucleus of a cell transfected with vAc^e18Δ29-38-gp^, in which clusters of microvesicles were produced along the nuclear membrane. In some regions, microvesicles existed between the outer and inner nuclear membranes (indicated by white triangles); in some regions, the inner nuclear membrane became blurred and could not be visualized, and the microvesicles were getting rid of the nuclear membrane or had already gone into the periphery region. As shown in Fig. 7C, OBs observed in the nucleus of a cell did not contain virus particles, and the surface of the OBs was densely covered with hair-like structures. Similarly, clusters of microvesicles were also observed along the nuclear membrane in the cells transfected with vAc^e18Δ39-48-gp^ (Fig. 7E and F). A few IMVs present near the nuclear membrane (Fig. 7F). Surprisingly, while there were some microvesicles budding into the nucleoplasm to form IMVs, there were also some microvesicles budding into the cytoplasm, in the same region of the nuclear membrane (Fig. 7A and E). Nucleocapsids were not found outside of the nuclear membrane or passing through the nuclear membrane in the cells transfected with either vAc^e18Δ29-38-gp^ or vAc^e18Δ39-48-gp^.

These results suggest that the TM domain of E18 is required for optimal formation and accumulation of IMVs and for intranuclear envelopment and nuclear egress of nucleocapsids; and the non-enveloped nucleocapsids could not be embedded into the OBs. The formation of the microvesicles budding into the cytoplasm from the nuclear membrane might be associated with the truncated E18 missing the TM domain, which resided outside of the nuclear membrane (Fig. 4C).

### The LCDs of E18 are essential for the intranuclear envelopment and nuclear egress of the nucleocapsids

**Fig 8:**
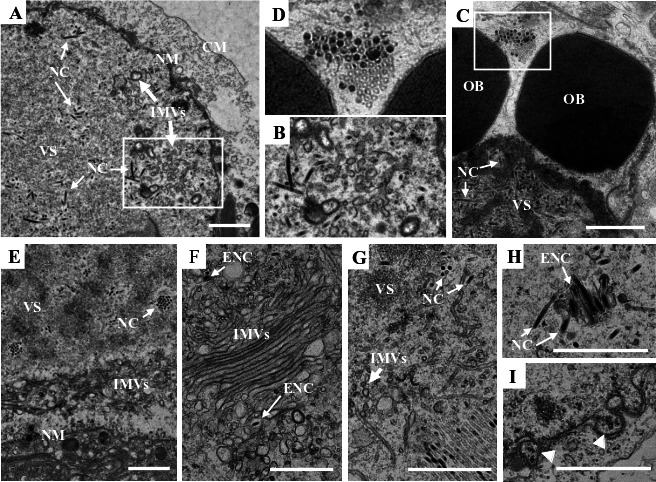
Transmission electron microscopy images of Sf9 cells transfected with vAc^e18Δ2-17-gp^ (**A–D**) or vAc^e18Δ49-59-gp^ (**E–I**), at 96 hpt. (**A, E**) Nucleocapsids within the VS and aggregated IMVs in the periphery region of the nucleus. (**B**) An enlargement of the outlined region in **A**, showing clusters of IMVs. (**C**) Two OBs without nucleocapsids, residing at the edge of the VS. (**D**) An enlargement of the outlined region in **C**, showing nucleocapsid/capsid aggregates. (**F**) A cluster of microvesicles containing some large, flat sacs, with a few enveloped nucleocapsids surrounded. (**G**) A cluster of nucleocapsids/capsids (lower right corner) and tubular structures (central part) at the edge of the VS. (**H**) A few putatively enveloped nucleocapsids. (**I**) Two nucleocapsids budding from the nuclear membrane (indicated by white triangles). CM, cytoplasmic membrane; NM, nuclear membrane; NC, nucleocapsid; VS, virogenic stroma; ENC, enveloped nucleocapsid. The white fine line arrows indicate the NCs or ENCs. The white thick arrows indicate the IMVs. Scale bar = 1 µm.

In cells transfected with vAc^e18Δ2-17-gp^ or vAc^e18Δ49-58-gp^, which lack the LCD1 and the LCD2 coding sequences of *e18,* respectively, virogenic stroma and nucleocapsids were observed in the nucleus (Fig. 8A, C, E and G). These cells contained numerous IMVs clustered into distinct aggregates (Fig. 8A, B, E and F), a pattern that contrasts with cells lacking the TM coding sequence, where such vesicles were distributed individually (Fig. 7A, E and F). In cells transfected with vAc^e18Δ2-17-gp^, no enveloped nucleocapsids were detected, nor was there any evidence of imminent nucleocapsid egress. As illustrated in Fig. 8C and D, a few suspected cross-sections of nucleocapsid/capsid aggregates were discernible at the periphery of the VS, potentially representing nucleocapsids containing nucleic acid or, conversely, empty capsids. For cells transfected with vAc^e18Δ49-58.gp^, a notable number of IMVs were densely packed in the ring zone, some of which coalesced to form expansive flat sacs (Fig. 8E and F). In Fig. 8G, a substantial cluster of nucleocapsids/capsids were observed at the edge of the VS. This area also featured numerous tubular structures, the nature of which—whether they represent deformed capsids or incomplete envelope precursors—remained uncertain. A small cluster of putatively enveloped nucleocapsids (ENC) was identified in a cell section within the ring zone (Fig. 8H). Furthermore, what appeared to be two nucleocapsids budding from the nuclear membrane is depicted in Fig. 8I (indicated by white triangles). Notably, the OBs documented in cells transfected with either vAc^e18Δ2-17.gp^ (Fig. 8C) or vAc^e18Δ49-58.gp^ were devoid of virions. These findings indicate that LCD1 plays a crucial role in the intranuclear envelopment of and the nuclear egress of nucleocapsids. Conversely, LCD2 is implicated in the accumulation and processing of IMVs and is indispensable for both intranuclear envelopment and nuclear egress of nucleocapsids.

### The AA18-28 of E18 is required for the multiply enveloped ODV formation

**Fig 9:**
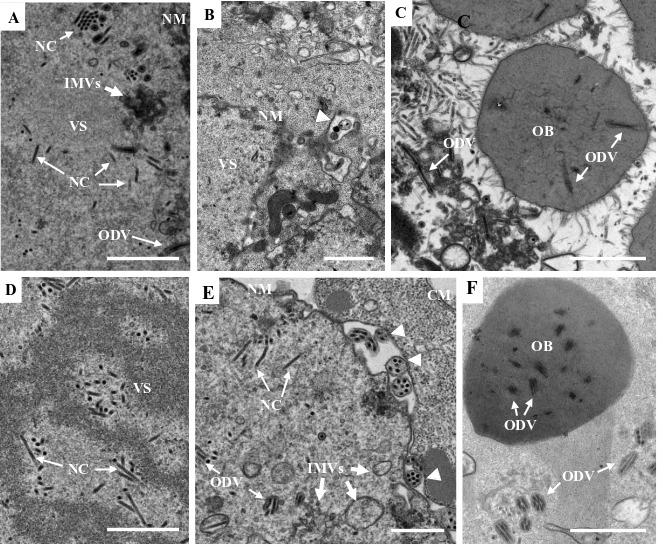
Transmission electron microscopy images of Sf9 cells transfected with vAc^e18Δ18-28-gp^ (**A–C**) or vAc^e18Δ70-79-gp^ (**D–F**) at 96 hpt. (**A**) Nucleocapsids within the VS and IMVs (indicated by white thick line arrows) and ODVs residing in the periphery region near the nuclear membrane. (**B**) Enveloped nucleocapsids within membrane vesicles outside of the nuclear membrane. (**C**) An OB with a few singly enveloped ODVs embedded and some singly enveloped ODVs around the OB. (**D**) Nucleocapsids within the VS. (**E**) Enveloped nucleocapsids residing between the outer nuclear membrane and inner nuclear membrane (indicated by white thick line arrows), scattered and aggregated IMVs, ODVs, and nucleocapsids in the periphery region of the nucleus. (**F**) An OB with multiple multiply or singly enveloped ODV virions embedded and a group of multiply enveloped ODVs near the OB. CM, cytoplasmic membrane; NM, nuclear membrane; NC, nucleocapsid; VS, virogenic stroma; ENC, enveloped nucleocapsid. The white fine line arrows indicate the NCs or ODVs. The white thick line arrows indicate the IMVs. Scale bar = 1 µm.

In the cells transfected with vAc^e18Δ18-28-gp^ or vAc^e18Δ70-79-gp^, several significant features were observed within the nucleus. These included typical virogenic stroma (Fig. 9A and D), aggregated IMVs (Fig. 9A and E), enveloped nucleocapsids (Fig. 9A, C, E and F), and OBs with embedded virions (Fig. 9C and F). In these cells, nucleocapsids encapsulated within membrane vesicles were apparent, either situated between the outer and inner nuclear membrane or in the cytoplasm (Fig. 9B and E). The OBs observed in vAc^e18Δ18-28-gp^-transfected cells contained only a few virions, each with a single nucleocapsid, and their surface displayed flagellated material attachments (Fig. 9C). Conversely, the OBs in the cells transfected with vAc^e18Δ70-79-gp^ contained numerous virions, each with one or more nucleocapsids, and their surface was smooth (Fig. 9F). These observations suggest that the AA70-79 region of E18 does not have a significant effect on ODV production, embedding of ODVs into the OBs, and the nuclear egress of the nucleocapsids, while the AA18-28 likely has a subtle influence on the nucleocapsid envelopment, associating with the formation of the multiply enveloped ODVs, and may also impact the ODV occlusion.

### Impact of E18 segment truncation on BV reproduction

**Fig 10:**
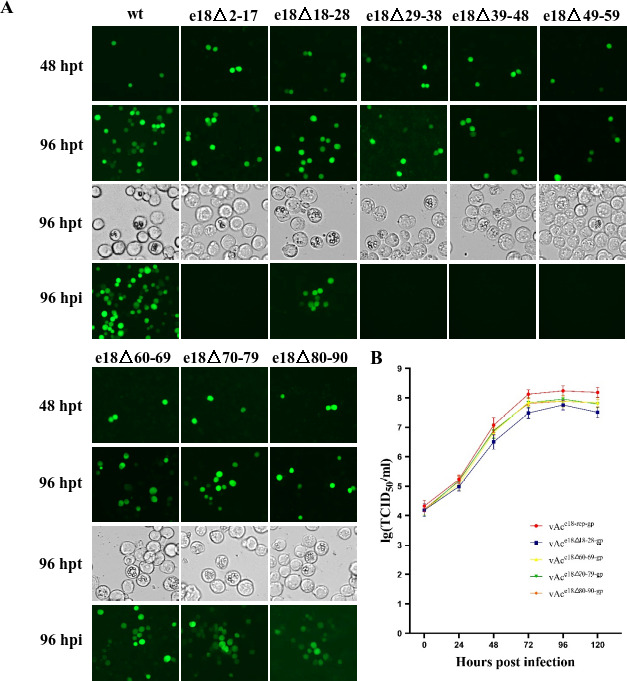
The analysis of BV production in Sf9 cells transfected with AcMNPV recombinants containing truncated *e18*. (**A**) Sf9 cells transfected with vAc^e18ko-rep-gp^ (e18ko-rep), vAc^e18Δ2-17-gp^ (e18Δ2-17), vAc^e18Δ18-28-gp^ (e18Δ18-28), vAc^e18Δ29-38-gp^ (e18Δ29-38), vAc^e18Δ39-48-gp^ (e18Δ39-48), vAc^e18Δ49-59-gp^ (e18Δ49-59), vAc^e18Δ60-69-gp^ (e18Δ60-69), vAc^e18Δ70-79-gp^ (e18Δ70-79), and vAc^e18Δ80-90-gp^ (e18Δ80-90) were subjected to fluorescence microscopy at 48 and 96 hours post-transfection (hpt) and light microscopy at 96 hpt. Following this, fresh Sf9 cell cultures were inoculated with the supernatant from these specific transfections and further examined by fluorescence microscopy at 96 hours post-infection (hpi). (**B**) BV growth curves of vAc^e18ko-rep-gp^, vAc^e18Δ18-28-gp^, vAc^e18Δ60-69-gp^, vAc^e18Δ70-79-gp^, and vAc^e18Δ80-90-gp^ in Sf9 cell cultures. Sf9 cell cultures were infected with the specific viruses at an MOI of 1. The supernatants were harvested at designated time points, and the virus titers were determined by TCID50 end-point dilution assays. Each data point represents the average titer of three independent infections. Error bars indicate standard deviations.

To assess the impact of various E18 segment truncations on BV replication, we conducted transfection-infection assays using the aforementioned *e18* truncation bacmids. In all experiments, the presence of green fluorescent cells at 48 hpt, as observed under a fluorescence microscope, confirmed successful bacmid introduction into the cells and subsequent viral gene expression. By 96 hpt, dishes containing vAc^e18ko-rep-gp^ exhibited fluorescence and OBs in most cells. Notably, there was a pronounced increase in fluorescent cells in dishes with vAc^e18Δ18-28-gp^, vAc^e18Δ60-69-gp^, vAc^e18Δ70-79-gp^, and vAc^e18Δ80-90-gp^. Conversely, a more modest rise was observed in dishes transfected with vAc^e18Δ2-17-gp^, vAc^e18Δ29-38-gp^, vAc^e18Δ39-48-gp^, or vAc^e18Δ49-59-gp^ (Fig. 10). No fluorescence was detected in dishes treated with supernatants from transfections involving vAc^e18Δ2-17-gp^, vAc^e18Δ29-38-gp^, vAc^e18Δ39-48-gp^, or vAc^e18Δ49-59-gp^, suggesting the absence of infectious BV production with these four bacmids. However, dishes receiving supernatants from the vAc^e18Δ18-28-gp^, vAc^e18Δ60-69-gp^, vAc^e18Δ70-79-gp^, or vAc^e18Δ80-90-gp^ transfections displayed fluorescent cells, though their numbers were significantly lower compared to the vAc^e18ko-rep-gp^ dish (Fig. 10A). These results correspond to the phenotype revealed by electron microscopic analysis that the nuclear egress of the nucleocapsids was not seen in the cells transfected with vAc^e18Δ2-17-gp^, vAc^e18Δ29-38-gp^, and vAc^e18Δ39-48-gp^ (Fig. 7 and 8), whereas it was observed clearly in the cells transfected with vAc^e18Δ18-28-gp^, vAc^e18Δ70-79-gp^, and vAc^e18ko-rep-gp^ (Fig. 2B, 9B and E) since the nuclear egress of the nucleocapsids is a prerequisite for BV production. Figure 10B shows the growth curves of vAc^e18Δ18-28-gp^, vAc^e18Δ60-69-gp^, vAc^e18Δ70-79-gp^, and vAc^e18Δ80-90-gp^ in Sf9 cells, infected at an MOI of 1. The BV titers of vAc^e18ko-rep-gp^, at all of the time points for detection, were higher than those of the other four bacmids with a truncated *e18*. The peak titers of vAc^e18ko-rep-gp^, vAc^e18Δ18-28-gp^, vAc^e18Δ60-69-gp^, vAc^e18Δ70-79-gp^, and vAc^e18Δ80-90-gp^, reaching at 96 hpi, were 1.7 × 10^8^, 5.8 × 10^7^, 7.8 × 10^7^, 9.1 × 10^7^, and 7.8 × 10^7^ TCID_50_ unit per milliliter, respectively. This indicates that while these recombinant bacmids with truncated *e18* supported the reproduction of infectious BVs, the deletion of aa18-28, aa60-69, aa70-79, or aa80-90 resulted in reduced BV yield.

These findings indicate that both the TM domain and the two LCDs of E18 are essential for the production of BV. Furthermore, the regions spanning amino acid sequences AA2-28, AA60-69, and AA70-79 to AA80-90 have been identified as influencing BV productivity.

## DISCUSSION

In this study, we established that AcMNPV *e18* is essential for ODV production. Our findings reveal that the deletion of *e18* from the AcMNPV genome decreases the formation of IMVs, blocks the accumulation of IMVs, nuclear envelopment and nuclear egress of nucleocapsids, and the incorporation of ODV into OBs during virus morphogenesis. Similar phenotypes were observed in AcMNPV recombinants lacking *p48*, *ac75*, *ac76*, *ac93*, or *ac106*. The removal of each of these genes led to a defect in IMV formation and subsequent envelopment of nucleocapsids to form ODVs and embedding of ODVs into OBs (10, 15, 26–30). It is evident that naked nucleocapsids cannot be embedded into OBs. Notably, the nuclear egress of the nucleocapsids was also inhibited by deleting any of the aforementioned genes. The nuclear egress of the nucleocapsids appears to be an event distinct from the envelopment of the nucleocapsids to form ODVs, although both processes involve interactions between the nucleocapsids and membranes. The IMVs, which serve as precursors to the ODV envelope, are derived from the viral protein-modified nuclear membrane (16, 17, 19, 28, 31). The nuclear egress of the nucleocapsids is also accomplished by budding through the modified nuclear membrane (2, 32, 33). Therefore, it is reasonable to assume that the same set of viral proteins that are involved in modifying the nuclear membrane and participating in the formation of IMVs and ODV nucleocapsid envelopment also mediate the nuclear egress of nucleocapsids. The correlation between intranuclear envelopment and nuclear egress of nucleocapsids was confirmed by the phenotypic differences observed in the TM and LCD deletion mutants in this study. The absence of TM or LCD1 resulted in a lack of envelopment of nucleocapsids, thereby preventing their exit from the nucleus. Enveloped nucleocapsids were occasionally observed in cells transfected with the LCD2-null mutant, and budding nucleocapsids from the nuclear membrane were also detected. According to previous studies, intricate interactions exist between AC75, AC76, AC93, AC106, and P48. AC106 could interact with AC75, AC76, and AC93. AC76, AC93, and P48 are interconnected, while AC75 exhibits associations with AC76 and AC93 (10, 28, 29, 34, 35). It has been hypothesized that these five proteins may form a complex to play roles in ODV envelopment (28). If this complex indeed exists, E18 could be the sixth associated protein. In earlier research, E18 was observed to associate with IMVs (22) and interact with AC76, P48, and two other proteins ODV-E25 and AC132, which are also involved in ODV envelopment (35, 36).

In order to further delineate the functional domains of E18, we constructed and analyzed a series of AcMNPV recombinant bacmids containing mutated *e18* with varying lengths of truncated amino acid-encoding sequences. These were compared to wild-type and *e18* knockout repair mutants. Given that processes such as envelopment, nucleocapsid nuclear egress, and ODV occlusion occur within the nucleus, we investigated the impact of these truncations on E18’s trafficking into the nucleus. The trafficking of ODV-E66 and several other ODV proteins from the ER to the inner nuclear membrane is facilitated by the N-terminal INM-SM domain, with positively charged amino acids playing a crucial role in membrane integration via interaction with cellular importing–α and viral proteins FP25 and BV/ODV-E26 (17–20, 37, 38). Another subset of ODV envelope proteins, which includes PIF5, PIF6, Ac76, Ac78, Ac108, F-protein, and E18, has their transmembrane domain situated toward the middle or near the C-terminal end. These are accompanied by positively charged amino acids proximate to the N- and/or C-terminal ends of the TM domain. Such proteins are categorized as atypical INM-SM (21). E18 features a hydrophobic transmembrane domain (aa27-48) positioned centrally and a positively charged arginine located immediately upstream of the TM (at a distance of three amino acids). Our findings indicate that all truncations outside the TM domain did not affect the nuclear trafficking of E18 significantly. Only AA30-34 within the TM was necessary for its nuclear import, but it was not sufficient to mediate the nuclear import of a fusion protein. Since E18 can only enter the nucleus in the presence of other viral proteins, as previously reported and confirmed in this study, AA30-34 may function as the binding site for the carrier protein responsible for its nuclear delivery. Our findings also reveal that the intact α-helix structure, which includes the TM domain, can effectively facilitate the trafficking of the fusion protein into the nucleus. Conversely, the positively charged arginine (R24) adjacent to the TM does not appear to impact the nuclear import of E18. These insights suggest that the nuclear import pathway of E18 differs from that of ODV-E66. Unlike ODV-E66 and most PIF proteins, E18 is shared between ODV and BV. This implies that a portion of the E18 synthesized on the ribosomes is directed into the nucleus, while another portion may be routed to the cell membrane, where BVs bud and obtain their envelope. There should be an as-yet-undetermined distribution mechanism at play that could also influence its nuclear transport. Notably, much like E18, ODV-E25 and the majority of other envelope proteins common to both ODV and BV contain atypical INM-SM (21). The nuclear trafficking mechanisms of these proteins are likely distinct from those mediated by typical INM-SM, a topic warranting further exploration.

Electron microscopy analysis revealed that both the TM and the LCDs of E18 are essential for the intranuclear envelopment of the nucleocapsids. These elements likely operated at distinct stages in the processing of IMVs into unit membranes and/or the nucleocapsid coating. The absence of TM led to a reduction and nonclustering of IMVs, whereas the absence of LDC2 promoted accumulation and fusion of a large number of IMVs. LCDs are sequences in proteins characterized by a compositional bias, often enriched in charged or polar amino acid residues, sometimes presenting as single or limited amino acid repeats, and typically lacking secondary and tertiary folding structures (39). Such LCDs contribute to binding diversity (40). Researchers have identified a wide array of functional roles for LCDs, including sites for posttranslational modifications; binding sites for DNA, rRNA, mRNA, tRNA, metal ions, and other proteins; regulatory protease digestion sites; signals for nuclear localization; flexible linkers between structured domains; and mediators of cellular regulation (41). Many virus proteins feature LCDs (39, 41); for instance, all tegument proteins of Herpes simplex virus 1 (HSV-1) contain LCDs, with one particular tegument protein, UL11, undergoing liquid-liquid phase separation *in vitro* (42). A short linear motif in the LCD of AcMNPV IE1 was demonstrated to be necessary for the coordinated localization of IE1 within the nucleus (43). E18 possesses two LCDs with distinct amino acid compositions. Omitting either one from E18 results in IMVs remaining in an aggregated state, though a few enveloped nucleocapsids were observed in cells transfected with the LCD 2-null mutant. Nevertheless, the phenotypic alterations induced by the absence of each of the two LCDs varied (Fig. 8), suggesting that these LCDs might serve as binding sites for other proteins involved in processing IMVs into unit membranes and/or the nucleocapsid coating, potentially interacting with different proteins. Furthermore, there is uncertainty regarding whether the substantial clusters of empty capsids observed at the VS edges in cells transfected with bacmids devoid of LCD 1 or LCD 2 coding sequences are indicative of any abnormalities in liquid-liquid separation. These abnormalities could potentially arise from the absence of one or both of the LCDs. The sequences containing LCD 1 and LCD 2 of E18 are conserved in most Group I alphabaculoviruses (Fig. 5 in reference 14). In addition to the TM and two LCDs, AA18-28 was also found to play a role in virus morphogenesis by influencing formation of multiply enveloped ODV virions. A similar phenotype was reported previously for an *ac92* knockout mutant (9). Unlike the *ac92*-null mutant, which lost infectivity, the *e18* aa18-28 truncation mutant was able to produce infectious BV (Fig. 10).

In addition to the morphogenesis occurring within the nucleus, the production of BV involves the nuclear egress of the nucleocapsids, the passage of the nucleocapsids through the cytoplasm to the cell membrane, and the budding of the nucleocapsids from the cell membrane. The experimental results from this study demonstrated that *e18* played a crucial role in the nuclear egress of nucleocapsids, thereby impacting the production of BV. Other viral genes that have been identified to involve the nuclear egress of the nucleocapsids include *gp41, ac75, ac76, ac93, ac106, p48, ac66, ac141*, *vubi*, *ac11, ac13,* and *ac51* (10, 21, 26–30, 44–49). It was shown that AC66, a BV nucleocapsid protein, underwent ubiquitination by vUbi, with interactions between AC141 (a potential E3 ubiquitin ligase), AC66, and vUbi being essential for nucleocapsid egress (45). Elevated ubiquitination levels of BV nucleocapsids were speculated to be a signal for nucleocapsid egress. Furthermore, the endosomal sorting complex required for transport (ESCRT) III of *Spodoptera frugiperda* was found to be required for the nuclear egress of the AcMNPV nucleocapsid egress (50). A recent study revealed that E18, along with 15 other viral proteins (AC17, AC75, AC76, AC78, AC93, AC146, Exon0, ODV-E25, GP41, ME53, P12, P48, PTP, TLP, and vUbi), interacted with all ESCRT-III components, creating a complex interaction network with each other or with other viral proteins, by Y2H and BiFC analysis, although the authors did not include E18 in their proposed nucleocapsid egress complex (35). The mechanism by which E18 facilitates nucleocapsid egress warrants further investigation. Given its location within the BV envelope, E18 may also play a role in the budding process of nucleocapsids from the plasma membrane. In the aforementioned report, E18 was included in a predicted BV entry and budding complex as ESCRT-III is also involved in both BV entry and budding (35). Furthermore, another study demonstrated that BmNPV E18 interacts with GP64, a major BV envelope protein crucial for BV entry and efficient budding (51–53). In this study, we noted a significant decrease in BV production when AA18-28, AA60-69, AA70-79, or AA80-90 segments were removed from E18. However, as the extraction analysis of BV released from infected cells was not conducted, it remains unclear whether these specific segments contain functional domains related to BV budding or invasion.

## MATERIALS AND METHODS

### Virus, cell line, and primers

The wild-type virus utilized in this research is AcMNPV (E2). The Sf9 cell line, a clonal derivative of the parent cell line IPLB-Sf21-AE, was originally derived from the fall armyworm *Spodoptera frugiperda* (54). These cells were cultured at 27°C using Grace’s medium (Invitrogen Life Technologies), which was supplemented with 10% fetal bovine serum, penicillin (100 µg/mL), and streptomycin (100 µg/mL).

The DNA primers used in the experiments are listed in Table 1.

**TABLE 1:** Oligonucleotides used in this study^*a*^

Primer	Sequence (5’−3’)
gfpPF	GTGAGCAAGGGCGAGGAGCTG
gfpPR	TTACTTGTACAGCTCGTCCATG
pFBD-gfp-ph-F	GCCGCATCGTTGCTATGAGAATTCCTATGAACGCGTTCTAC
Pe18UF	GCCGCATCGTTGCTATGAGAATTCCTATGAACGCGTTCTAC (nt 124809–124826)
Pe18DR	AGGTGAACTGTTGTAGCGGAATTCCTTTGTTGCATGTG (nt 125440–125454)
Pe18Δ2-28UR	AGCCAAGATGGTCAAGAACATATTATTGTACCGAGTCGGGGAT (nt 125246–125226/125141–125120)
Pe18Δ2-28DF	ATGTTCTTGACCATCTTGGCT (nt 125226–125247)
Pe18Δ25-48UR	AGCTGTTTCCATTACTGCTAGACCTGTTTAAATAGTTTGTGGACG (nt 125307–125286/125213–125191)
Pe18Δ25-48DF	TCTAGCAGTAATGGAAACAGCT (nt 125286–125307)
Pe18Δ49-90UR	TCATTTTTTATACACTTATCTATTGAACAAATATAATTATTAAAGC (nt 125433–125412/124285–125262)
Pe18Δ49-90DF	TAGATAAGTGTATAAAAAATGA (nt 125412–125433)
Pe18Δ2-17UR	CTGTTTAAATAGTTTGTGGACATATTATTGTACCGAGTCG (nt 125212–125193/125144–125125)
Pe18Δ2-17DF	TCCACAAACTATTTAAACAG (nt 125193–125212)
Pe18Δ18-28UR	AGCCAAGATGGTCAAGAACATCGGCGCGTCTGTGCTAGT (nt 125242–125226/125192–125175)
Pe18Δ18-28DF	ATGTTCTTGACCATCTTGGCT (nt 125226–125246)
Pe18Δ29-38UR	ACAAATATAATTATTAAAGCAATAATGTTTGGAGTTAGCCTGTTTA (nt 125281–125256/125227–125206)
Pe18Δ29-38DF	ATTATTGCTTTAATAATTATATTTGT (nt 125256–125281)
Pe18Δ39-48UR	AGCTGTTTCCATTACTGCTAGATACTACTACAGCCAAGATGGTC (nt 125308–125286/125255–125234)
Pe18Δ39-48DF	TCTAGCAGTAATGGAAACAGCT (nt 125286–125308)
Pe18Δ49-59UR	AGGGCGTTTGGAGGTACTTGAACAAATATAATTATTAAAGC (nt 125335–125319/125285–125262)
Pe18Δ49-59DF	GTACCTCCAAACGCCCT (nt 125319–125335)
Pe18Δ60-69UR	CATGGTAGCGTTTAAAGGATTATTACCCCCCGAGCTGTTTC (nt 125369–125349/125318–125299)
Pe18Δ60-69DF	AATCCTTTAAACGCTACCATGCGA (nt 125349–125372)
Pe18Δ70-79UR	AGGCGTGTTCATAAAGGGTACAAAACCCCCCAGGGC (nt 125397–125379/125348–125331)
Pe18Δ70-79DF	CCCTTTATGAACACGCCT (nt 125379–125397)
Pe18Δ80-90UR	TCATTTTTTATACACTTATCTAATTAGCTCGCATGGTAGCGT (nt 125433–125412/125378–125359)
Pe18Δ80-90DF	TAGATAAGTGTATAAAAAATGA (nt 125412–125433)
Pe18Δ25-42UR	CCTGTTTAAATAGTTTGTGGACG (nt 125213–125191)
Pe18Δ25-42DF	CGTCCACAAACTATTTAAACAGGATAATTATATTTGTTCAATCTAGC (nt 125191–125213/125268–125291)
Pe18Δ25-34UR	CCTGTTTAAATAGTTTGTGGACG (nt 125213–125191)
Pe18Δ25-34DF	CGTCCACAAACTATTTAAACAGGGCTGTAGTAGTAATTATTGCT (nt 125191–125213/125244–125264)
Pe18Δ35-44UR	CAAGATGGTCAAGAACATGT (nt 125243–125224)
Pe18Δ35-44DF	ACATGTTCTTGACCATCTTGATATTTGTTCAATCTAGCAG (nt 125224–125243/125274–125293)
Pe18Δ29-34UR	GTTTGGAGTTAGCCTGTT (nt 125225–125208)
Pe18Δ29-34DF	AACAGGCTAACTCCAAACGCTGTAGTAGTAATTATTGCT (nt 125208–125225/125244–125264)

^*a*^ The numbers in parentheses represent the genomic positions of the primer sequences; where two sets of values are given, the underlined value represents the position of the underlined sequence.

### Preparation of polyclonal antibodies against *e18*

The AcMNPV *e18* ORF, with EcoR I and Sal I sites added at its 5’- and 3’-end, respectively, was PCR-amplified and cloned into the corresponding sites of the pET28a plasmid (Invitrogen), resulting in the construct pET-e18. *E. coli* BL21(DE3) pLysS cells transformed with pET-e18 were induced using IPTG, leading to the expression of the His-tagged E18 protein. This protein was subsequently purified utilizing Ni-NTA resin (Qiagen). After purification, the eluate underwent SDS-PAGE, and the band corresponding to the His-tagged E18 protein was isolated from the gel, homogenized, and used for mouse immunization. Two weeks post-initial inoculation, the mouse received three additional booster injections at 2-week intervals. Ten days following the final boost, the mouse was bled, and the resultant serum was preserved for subsequent use in this study.

### Transfection and infection

To transfect Sf9 cells in culture, they were seeded into 35-mm dishes at a density of 1.0 × 10^6^ cells/dish and incubated overnight at 27°C. Subsequently, 2 μg of bacmid was combined with 5 μL of FuGene HD transfection reagent (cat. no. E2311, Promega). This mixture was then diluted to 100 μL using the growth medium and added to the dishes. The dishes were gently mixed and incubated for designated durations. For infection experiments, 1 × 10^6^ Sf9 cells in 35-mm dishes were incubated at 27°C overnight. The medium was then discarded, and either 10 μL of the infectious supernatant from a wild-type bacmid transfection or 500 μL from a recombinant bacmid transfection (all supernatants collected at 120 hpt) was added, and incubation was continued for the desired time period. For titering of infectious BV, Sf9 cells in 35-mm dishes were inoculated with the supernatant from cultures with individual recombinant virus, at an MOI of 1. The supernatants were collected at 0, 24, 48, 72, 96, and 120 hpi. The BV titers were determined using a TCID50 end-point dilution assay (55). Virus infection was determined by viewing green fluorescence from EGFP expressed by the viruses.

### Immunofluorescence assays and confocal microscopy

Immunofluorescence assays and confocal microscopy were conducted as previously described (36). Briefly, Sf9 cells were seeded onto the surface of coverslips in 35-mm dishes. These cells were then inoculated with the infectious supernatant of AcMNPV or transfected with individual bacmids, as detailed below. At specified time points post-infection or post-transfection, the cells on the coverslips were fixed using an immunofluorescence fix solution (Beyotime). Subsequently, they were incubated with an E18-specific antibody, followed by incubation with Rhodamine (TRITC)-conjugated goat-anti-mouse IgG (PTG Lab) at a dilution of 1:60. The cells were then stained with Hoechst 33258 (Beyotime). After being sealed on microscope slides with antifade mounting medium (Beyotime), the samples were subjected to confocal microscopic analysis using a confocal laser scanning microscope to detect fluorescence.

### Plasmid and bacmid construction

A fragment containing the *egfp* ORF was PCR-amplified using primers gfpPF and gfpPR, excluding the start codon. This fragment was subsequently inserted between the Xho I and Mlu I sites of pIZ/V5-His (Invitrogen) to create pIZ-gfp. Then, a PCR-amplified fragment containing the *e18* coding sequence excluding the termination codon was inserted upstream of the *gfp* of pIZ-gfp, using the pEASY-basic seamless cloning and assembly kit, making pIZ-e18.gfp.

The bMON14272 bacmid, originally derived from the AcMNPV strain E2, was maintained within DH10B cells as previously documented (56). The *e18* deletion from the AcMNPV genome was executed utilizing the λ Red system, as detailed in previous literature (57). In summary, two DNA fragments, corresponding to nt 124272–125152 and nt 125216–125781 of the AcMNPV genome, were separately amplified via PCR and integrated between the Xba I and BamH I, and the PstI and HindIII sites of the plasmid pKS-cat, respectively. Subsequently, the resulting plasmid was cleaved with Xba I and Hind III. The fragment containing the chloramphenicol acetyltransferase gene (cat) cassette, flanked by the viral sequences, was isolated and electroporated into arabinose-induced *E. coli* DH10B cells carrying bMON14272 and pKD46, which encode λ-Red recombinase. This yielded bacmid was designated as vAc^e18ko^ (Fig. 1A). The proper replacement of *e18* with the *cat* cassette within the bacmid was confirmed via PCR using four primer sets: uhUP/dhDP, uhUP/catDP, catUP/dhDP, and catUP/catDP.

A fragment containing *egfp*, under the control of the *gp16* promoter of AcMNPV, was PCR-amplified using the intermediate plasmid pFD-Pgp16-gfp-ie0. This fragment was inserted between the Xho I and EcoR I sites of pFD-ac75-PH (30), replacing the *ac75* sequence to create pFD-Pgp16-gfp-ph. This plasmid was then electroporated into *E. coli* DH 10B, which contained bMON14272 and a helper plasmid pMON7124 (56), to generate a *polh*- and *gfp*-containing wild-type bacmid vAc*^-p^* (Fig. 1A). Similarly, pFD-Pgp16-gfp-PH was electroporated into *E. coli* DH10B containing vAc^e18ko^ and pMON7124, resulting in a *gfp*- and *polh*-containing *e18*-knockout bacmid vAc^e18ko-gp^ (Fig. 1A). A fragment containing *e18* with its native promoter (333 bp upstream of the start codon ATG and 40 bp downstream of the stop codon) was amplified using primers E18UP and E18DP. This fragment was ligated with pFD-Pgp16-gfp-ph, cut with EcoR I, using the pEASY-basic seamless cloning and assembly kit, to create pFD-gfp-e18-ph. This plasmid was then electroporated into *E. coli* DH10B containing vAc^e18ko^ and pMON7124, generating the *e18*-repaired bacmid vAc^e18ko-rep-gp^ (Fig. 1A).

The bacmids containing truncated *e18* were constructed as follows: The *e18* upstream forward primer (Pe18UF) and downstream reverse primer (Pe18DR) were paired with Pe18Δ2-28UR and Pe18Δ2-28DF, respectively. This amplified an *e18* upstream fragment and an *e18* downstream fragment using pFD-gfp-e18-ph as a template. These fragments were then ligated with pFD-Pgp16-gfp-ph cut with EcoR I using the pEASY-basic seamless cloning and assembly kit, resulting in the transfer vector pFD-gfp-e18Δ2-28-ph. This was subsequently electroporated into *E. coli* DH10B containing vAc^e18ko^, producing vAc^e18Δ2-28-gp^ with a truncated coding sequence of AA2-28. Similarly, Pe18UF was paired with other -UR primers, and Pe18DR was paired with other -DF primers, as listed in Table 1. This amplified various *e18* upstream and downstream fragments, facilitating the construction of different transfer vectors and bacmids vAc^e18Δ25-48-gp^, vAc^e18Δ49-90-gp^, vAc^e18Δ2-17-gp^, vAc^e18Δ18-28-gp^, and others.

The bacmids containing a truncated *e18.gfp* fusion gene were constructed in the following manner: A fragment termed e18aa29-34.gfp, comprising the *e18* promoter and *e18* aa29-34 coding sequences with XhoI and PstI sites appended at the 5' and 3' ends, respectively, was synthesized by Qingke Company. This synthetic fragment was integrated into pFD-Pgp16-gfp-ph between the XhoI and PstI sites through homologous recombination, employing the pEASY-basic seamless cloning and assembly kit (Transgen), resulting in the creation of the transfer vector pFD-gfp-e18aa29-34.gfp. This vector was subsequently electroporated into *E. coli* DH10B carrying vAc^e18ko^ to produce vAc^e18aa29-34.gfp^. In an analogous fashion, synthetic fragments e18aa27-38.gfp, e18aa27-48.gfp, e18aa27-54.gfp, e18.gfp, and Pe18.gfp were utilized to generate transfer vectors, leading to the formation of bacmids vAc^e18aa29-34.gfp^, vAc^e18aa27-38.gfp^, vAc^e18aa27-48.gfp^, vAc^e18aa27-54.gfp^, vAc^e18.gfp^, and vAc^Pe18.gfp^, respectively.

All transfer vectors mentioned previously were sequenced to verify their construction. All bacmid constructs created were confirmed by PCR following the protocol in the Invitrogen Bac-to-Bac system manual.

### Electron microscopy

A total of 1 × 10^6^ Sf9 cells per dish (35 mm) were transfected with 1.0 µg of either vAc^e18ko-rep-gp^ or vAc^e18ko-gp^. At specified time intervals, the cells were fixed, subsequently dislodged, and then centrifuged at 3,000 rpm for a duration of 5 minutes. The resultant cell pellets underwent dehydration, embedding, sectioning, and staining, as detailed previously (58). The specimens were observed using a FEI Tecnai G2 20 TWIN transmission electron microscope operated at an accelerating voltage of 200 kV.

## Data Availability

All data related to this study are available in the paper.
